# Physical activity change after a promotora-led intervention in low-income Mexican American women residing in South Texas

**DOI:** 10.1186/s12889-019-7105-6

**Published:** 2019-06-20

**Authors:** Jennifer J. Salinas, Deborah Parra-Medina

**Affiliations:** 1 Department of Family Medicine, Texas Tech University Health Sciences Center El Paso, Paul L. Foster School of Medicine, Center of Emphasis in Cancer Research, 9849 Kenworthy St, El Paso, TX 79924 USA; 2 Latino Research Initiative, The University of Texas at Austin, 210 W. 24th Street, Mailcode F9200, GWB 2.102, Austin, Texas 78712 USA

**Keywords:** Mexican American, Women, Physical activity, Sedentary, Randomized control trial

## Abstract

**Background:**

The purpose of this study was to determine physical activity (PA) preferences associated with increases in moderate-to-vigorous physical activity (MVPA) and decrease in sedentary time in Mexican American (MA) women participating in a *Promotora* (community health worker)-led intervention on the U.S.-Mexico border.

**Methods:**

Enlace (‘to link’ in Spanish) was a randomized clinical trial to increase PA in low-income, MA women living in South Texas on the U.S.-Mexico border. A total of 620 participants were recruited into the study. The primary outcome was increase in moderate to vigorous physical activity (MVPA) using the Actigraph GT3X 16 Mb accelerometer. A modified version of the Community Health Activities Model Program for Seniors Physical Activity (CHAMPS) instrument was used to predict MVPA. Adjusted and unadjusted logistic regression models predicted change in MVPA by change in CHAMPS activities. ANOVA analysis determined the variance explained in change in MVPA by change in time engaged in activity. Individual effect sizes were then calculated for significant activity type change on MVPA increase.

**Results:**

There were significant increases in all CHAMPS activities except aerobic machines and errand walking. An increase in leisure walking (O.R. = 2.76, *p* = .046), errand (O.R. = 3.53, *p* = .051), and brisk walking (O.R. = 4.74, *p* = .011), dance (O.R. = 8.22, *p* = .003), aerobics class (O.R. = 32.7, *p* = .001), and light housework (O.R. = 6.75, *p* = .000), were associated with a decrease in sedentary time. Significant effect sizes for MVPA were observed for jogging (1.2, *p* = .050), general exercise (1.6, *p* = .024), and other exercise not specified (2.6, *p* = .003). Significant effect sizes for sedentary time were detected for leisure time (.031, *p* = .036), errands (.017, *p* = .022), brisk walking (.022, *p* = .003), dance (.042, *p* = .005), and aerobics class (.013, *p* = .009).

**Discussion:**

Participants who engaged in walking and aerobic activities through this intervention significantly increased their engagement in MVPA and decreased their sedentary time. These findings are novel, since preferences have not been examined in relation to MVPA or sedentary time in MA women.

**Conclusion:**

PA preferences need to be considered when aiming to promote activities that reduce sedentary time and increase PA participation among marginalized groups, such as MA women.

**Trial registration:**

NCT02046343.

## Background

Only 28.8% of Mexican American adults met the 2008 U.S. recommended guidelines for physical activity (PA) [1]. Mexican Americans also carry a high burden of chronic conditions, such as diabetes, obesity and uncontrolled hypertension [2]. Increasing PA engagement in this population will have substantial impact on reversing current chronic disease trends.

In response to low levels of PA across race/ethnic groups in the United States, some interventions have been implemented with mixed success [1, 3, 4]. One limitation has been the use of the recommended 150 min of moderate to vigorous PA as a targeted endpoint, as it is often unachievable or difficult to maintain over the long term for many [5–14]. With growing evidence of lighter intensity PA or simply shifting from sedentary to any activity having health benefits [6, 7], the 150 min of moderate to vigorous physical activity (MVPA) standard may not only be unachievable by most, it may not be the best strategy to improve health through meaningful behavioral change [15, 16].

Programs to increase PA in Mexican Americans have largely focused on children and families, thereby providing limited information on the effectiveness in women [17–24]. Evidence suggests that overall, Hispanic women tend to engage in less PA than men, and cultural barriers may play an important role in this disparity [25]. For example, Hispanic women in the Study of Latinos (SOL) engaged in 10 min less a day of physical activity, estimated via accelerometry [25]. Mexican American women may be more responsive to programs geared towards the cultural barriers that affect them the most, such as language, diet, or childrearing expectations. [26, 27]. However, culturally tailored programs related to energy balance only have been adapted linguistically or provided nutrition demonstrations or tips on ethnic dishes of the targeted group [28–30] Few, if any, have tailored PA interventions culturally to Mexican American or other Hispanic women, or have been sensitive to the needs of subpopulations like those living in rural settings or in extreme poverty [31, 32]. In many cases, these subpopulations are the least active and most at risk for chronic health conditions [33, 34].

Physical activity preference is an understudied area of research in general, but particularly in Hispanics subgroups [33, 34]. The limited information that is available suggests that preferences depend on age, gender, race/ethnicity and existing health conditions [5, 9, 34–40]. Among Hispanics, women are more likely to engage in PA around the home, whereas Hispanic men tend to prefer sport-based (e.g. soccer, football, etc.) activities [41]. Age is also an important factor, whereas, older adults prefer group-based activities (e.g. exercise classes, walking groups, etc.), while youth tend to be drawn to sports-based activities [40, 42]. However, while interventions should be adapted to these preferences, there is little information on whether preferences translate into meaningful change in PA levels. Moreover, little information is available on Mexican American subpopulations living in varying parts of the country where availability to PA resources may differ.

The current study makes use of data collected through a *Promotora* (community health worker) led PA intervention in South Texas on the U.S.-Mexico border region. Hidalgo County, the intervention site, is located on the southernmost tip of Texas and has the highest number of colonia settlements in the State of Texas [43, 44]. Colonias are unincorporated settlements that lack basic infrastructure such as electric and public sanitation [44]. Colonias in Hidalgo County are generally located in the most rural areas and are inhabited by Mexican Americans who live in extreme poverty. Physical activity participation in Hidalgo County is low with only 36.8% of adults engaging in any leisure time activity [45]. The intervention was tailored to the needs of women in this community recognizing the low baseline of PA engagement using community-based participatory research. In addition, the setting was largely rural, providing insight into a population that has been underrepresented in PA interventions target towards Hispanic populations. The setting and population allowed us to test research hypotheses on PA preferences and changes after an intervention in low income, rural-residing Mexican American women.

The purpose of this study was to assess changes in MVPA and sedentary time in low-income Mexican American women living in South Texas of the U.S.-Mexico border. We made use of multiple measures of subjective PA and sedentary time to evaluate which type of activities were most associated with intended change in the outcomes [46]. A notable strength of this study was the use of community-based participatory research where we engaged key stakeholders in an advisory committee and community members in focus groups to design the intervention. The education and physical activities were designed to address cultural barriers (e.g. gender roles, beliefs about health and wellness, etc.) and appeal to the preferences of our targeted population thereby reducing barriers (e.g. social control, low health literacy, among others.) to PA. As part of the education, we provided information on the recommendations for PA, health benefits and provide planned activities that are culturally sensitive and responsive to the resources available within the community. As a result of this approach, it was expected that increases in MVPA and decreases in sedentary time between pre and post intervention will be most associated with activities that were most sensitive to the community resources and barriers.

## Methods

### Enlace: a promotora-led physical activity intervention

This is a secondary data analysis of the Enlace study. Enlace was a 16 week-long promotora-led PA intervention randomized clinical trial (NCT02046343) funded by the National Heart, Lung and Blood Institute (NHLBI) of the National Institutes of Health (NIH) in the United States. A total of 8 community centers were randomized to either the physical activity intervention (PA) or control (community health and safety) using simple randomization because of the small n-size (8 centers). Centers were matched based on geographic location into pairs of two. Then were randomly assigned either as intervention or control group. While study staff knew which community centers were in the intervention or control conditions, participants were unaware of what the status was of other centers. The study was located in Hidalgo County of South Texas on the U.S.-Mexico border. Low-income, primarily immigrant, Mexican American women were recruited from 8 community resource centers (CRC’s) located in Cameron and Hidalgo Counties located in South Texas on the U.S.-Mexico border from 2012 until 2016. Four CRC’s were randomly assigned to receive the PA intervention (intervention) and four received a community health education program (control). Women were included if they did not meet the recommended level of PA of at least 150 min per week of MVPA. Women were excluded if they had a chronic condition (e.g. diabetes, hypertension, etc) and were not cleared by their doctors to participate or if they were pregnant. Women in the intervention group participated in weekly PA education sessions that were complemented by guided physical activities, such as, walking groups, Zumba® classes and yoga. The control group received a 16 week long community health and safety educational program without any other complementary activities. Each session lasted one to 2 h. Sample size for Enlace was calculated to reach a minimal detectable effect size for a power of .80. In total 620 women completed the baseline assessment and 439 completed follow-up. Reasons for intervention non-completion included securing employment, pregnancy, relocated outside of center catchment area, withdrew, non-compliant with accelerometry collection, and illness. About 20% of participants attended all sessions and about 50% attended most sessions (5 to 13) in the intervention group.

At baseline, women in the Enlace study were on average 40.4 +/− 10.3 years old (age range: 18–67 years), had an average of an 10th grade education and were primarily born in Mexico (86.1%) (See Table 1). Additionally, most were married or in a partner relationship (79.8%), only 23.1% were unemployed and 16.9% had health insurance. Significant differences between the intervention and control group were observed for body mass index (BMI) (kg/m2) only (intervention 32.8 vs. control 30.8, *p* = .039). There was, however, a near significant difference by age (intervention 39.7 years vs. control 41.2 years, *p* = .078). Therefore, these two significant variables were used as covariates in the regression analysis.

**Table 1:** Baseline demographic and anthropometric covariates by intervention status in the Enlace sample (*n* = 620)^a^

	Total	Intervention	Control	*P*-value
Demographics				
Age (mean ± s.d.)	40.4 (10.3)	39.7 (10.1)	41.2 (10.4)	.078
Years of education (mean ± s.d.)	9.8 (4.7)	10.0 (5.6)	9.6 (3.5)	.309
Country of birth (n(%))				
United States	80 (12.9)	40 (11.8)	40 (14.2)	.382
Mexico	534 (86.1)	296 (87.6)	238 (84.4)	
Other	6 (.97)	2 (.59)	4 (1.4)	
Marital status (n(%))				
Married/common law/partnered	495 (79.8)	272 (80.5)	223 (79.1)	.666
Not married	125 (20.2)	66 (19.5)	59 (20.9)	
Number of children living in household (mean ± s.d.)	2.5 (3.8)	2.7 (5.0)	2.4 (1.6)	.304
Employed (n(%))	143 (23.1)	82 (24.3)	61 (21.6)	.439
Health insurance (yes)(n(%))	104 (16.9)	54 (16.0)	50 (17.9)	.521
Baseline Anthropometrics				
Body mass index (mean ± s.d.)	31.9 (11.8)	32.8 (14.6)	30.8 (6.9)	.039
% Body Fat (mean ± s.d.)	39.9 (7.5)	40.1 (7.7)	39.5 (7.2)	.317
Waist circumference (mean ± s.d.)	100.0 (14.1)	100.7 (15.3)	99.2 (12.5)	.187

^a^two-tailed significance test

### Intervention description

Enlace participants engaged in a 16-week long intervention that included educational classes and promotora-led PA sessions (i.e. aerobic or walking groups). Classes were offered weekly and PA session were offered throughout the week. The PA group (intervention) were provided information on the health benefits of PA, what is considered PA, different types of PA, levels of intensity, PA guidelines, PA demonstrations and group exercise activities (e.g. exercise classes, DVD’s and walking groups). Promotoras also coordinated walking groups and exercise classes offered through the community centers. A 6-month maintenance period followed the intervention whereas promotoras met with participants individually every month and offered guided group-based physical activities such as Zumba® classes and walking groups. The control group received a community health and safety curriculum and were also offered activities during the maintenance period.

### Data and measures

#### Outcome variable

##### Moderate to vigorous physical activity (MVPA)

MVPA was measure using the ActiGraph GT3X 16 Mb accelerometer. Actigraphs were given to the participants at baseline and 16-week data collection interviews. At the end of each interval, the summed value or activity “count” was stored in memory and the integrator was reset. We determined the total time per day spent in MVPA and sedentary time by using adapted count cut points previously established by Freedson et al. [47, 48]. In general counts are based on frequency and intensity of acceleration. Sedentary activity was determined to be counts per minute of less than 99. Moderate activity counts per minute were 1952–5724 and vigorous 5725–9498. Freedson, et al. [47, 48] has documented the validity and inter-instrument reliability of the ActiGraph monitor in adults. Activity counts were strongly correlated with energy expenditure during treadmill walking and running (r = 0.86). Minutes for moderate and vigorous activity were combined to generate values for our MVPA outcome.

Participants were given accelerometers during their first data collection appointment. They were told to wear the device every day from the time they woke up until they went to bed. They were only to take it off to shower. On the second day of data collection, a week after the first visit, accelerometers were collected from participants and checked for wear time. If participants did not have adequate wear time, accelerometers were returned for re-wear. Participants needed to wear the accelerometer for at least 12 h per day and on at least 4 of the 7 days for reliable measurement of activity. Participants were also asked to wear the device on both weekdays and weekend days. Because average per day minutes were reported by the accelerometer, we converted minutes per day to minutes per week by multiplying by seven. The total minutes per week at baseline and 16-week follow-up were used for this analysis. We used intent to treat (ITT) to address missing outcomes at 16 week follow-up due to drop out from the study [49]. Missing values at 16 weeks were replaced with values from baseline. A categorical variable was created to measure an increase in MVPA between baseline and 16 week follow-up. The difference between follow-up MVPA and baseline MVPA was calculated and an increase was coded as “1”, otherwise “0” for values of zero or less. Similarly, a variable was created for sedentary time, however a decrease in sedentary time was coded as “1” and “0” for no change or an increase.

### Explanatory variables

#### Community health activities model program for seniors physical activity questionnaire (CHAMPS)

We used the CHAMPS to characterize participants’ type and amount of PA. Knowing which types of PA correspond most directly with increases in MVPA and decreases in sedentary time. The original 41-item self-reported measure of type of PA was developed originally for older adults, but it has been used with general adult populations and sensitive to changes in intervention studies [50, 51]. We removed questions that were not relevant to our targeted population For example, questions related to reading, playing a musical instrument, and shooting pool were not included in this study. The benefit of using CHAMPS over other instruments is that in the CHAMPS PA is assessed by an algorithm of different types of physical activities to calculate moderate, vigorous and leisure time activity. As a result we were able to determine to what extent our intervention impacts specific types of activities. Additionally, we were able to determine which activities corresponded most with changes in objectively measured MVPA. The CHAMPS asked participants to recall over the past month, typically how many hours per week do they engage in the listed activity. We used hours per week for each individual activity (e.g. dance, heavy gardening, yoga, walking, etc.). Differences between baseline and 16-week follow-up were calculated and a categorical variable was created. Increased activity of 30 min or more were coded as “1” for each of the 41 items. No change or a decrease in the amount of time spent was coded as “0”. The threshold of 30 min per week change was set because it was the lowest measureable unit in the instrument that was used for this study.

### Covariates

We did an initial analysis to assess for successful randomization and to determine if control variables would need to be adjusted for. The variables used were age (continuous), education (continuous), birth country (U.S., Mexico, other), marital status (married/not married), number of children (continuous), employment status (employed/not employed), and health insurance (yes/no). We also used body mass index (BMI), % body fat, and waist circumference to account for any variation in baseline obesity status that might affect intervention participation. Both BMI and % body fat was calculated using a portable Tanita Body Composition Analyzer SC-331S. Height (measured to the nearest 0.1 cm) was inputted manually for BMI calculations using a stadiometer. Participants were asked to remove their shoes prior to stepping on the Tanita and obtaining height measurements. BMI was calculated using the weight (kg)/height squared (m2) formula. Waist circumference was measured twice at the midway between the iliac crests and the lower ribs.

### Analysis

Initial analysis was conducted to establish the success of randomization. Chi-squared and t-test analyses were conducted by intervention status using the covariates. From this analysis, Body Mass Index (BMI) (*p* = .039) varied significantly by intervention group. In addition, age was near significant (*p* = .078). Therefore we used both variables as controls in the regression analysis. Chi-squared and t-test analyses were also conducted for each CHAMPS activity change bivariate variable and change in MVPA by intervention group. CHAMPS activities that were not significantly different were not used in the regression analysis. Additionally, variables from the bivariate analysis that were stretching or yoga were not analyzed in the logistic models due because focus was placed on variables most likely to move participants from sedentary movement to MVPA. Adjusted and unadjusted logistic regression models were then conducted to predict change in MVPA by change in significant CHAMPS activities. Odds ratios were reported for each activity for both adjusted and unadjusted models. ANOVA analysis was then conducted on the continuous change in MVPA outcome variable to determine the overall variance explained for the model that included all significant CHAMPS variables. Individual effect sizes were then calculated for each individual CHAMPS variable to determine the proportion of variance explained by each. Final analysis was only conducted on the intervention group to examine the within group effect of the exposure to the physical activity programming.

## Results

Table 2 presents increases in activity type by intervention status by category of activity. A significantly higher proportion of participants in the intervention group increased their overall walking, aerobic exercise and strength or stretching activities by at least 30 min per week between baseline and 16-week follow-up. For example, 43.5% of women in the intervention group reported increasing their leisure time walking by at least 30 min compared to 27.0% in the control group. It should be noted that as part of the intervention, women were offered aerobic and Zumba® classes, stretching, yoga and promotora-led walking groups. Additionally, 32.8% in the intervention group reported increasing their engagement in aerobic classes (primarily Zumba®) compared to 17.8% among the control group. Other significant differences were observed in heavy gardening (28.7% v. 12.8%), light housework (38.2% vs. 30.5%), general exercise (14.5% vs. 3.9%) and other not-specified activity (5.6% vs. 1.8%). There were no significant differences by intervention group for swimming activities or leisure activities such as golf, tennis or hiking. This is most likely due to the low SES status of our participants and a lack of amenities in the area for hiking.

**Table 2:** Any increase in hours per week activity type at post intervention by intervention status in the Enlace sample (*n* = 620)^*^

	Intervention	Control	*P*-value†
Walking			
Leisure time	147 (43.5)	76 (27.0)	.000
Errand	82 (24.3)	43 (15.2)	.005
Brisk	104 (30.8)	44 (15.6)	.000
Aerobic exercise			
Dance	87 (25.7)	27 (9.6)	.000
Aerobic class (Zumba®)	111 (32.8)	24 (17.8)	.000
Aerobic machines	26 (7.7)	8 (2.8)	.008
Jogging	67 (19.8)	18 (6.4)	.000
Lifestyle			
Light housework	129 (38.2)	86 (30.5)	.046
Heavy housework	79 (23.4)	48 (17.0)	.051
Light gardening	105 (31.1)	69 (24.5)	.069
Heavy gardening	97 (28.7)	36 (12.8)	.000
Swimming			
Water exercises	7 (2.1)	1 (.35)	.059
Moderate Swimming	7 (2.1)	1 (.35)	.059
Slow Swimming	8 (2.4)	2 (.71)	.103
Stretch/Strength			
Yoga	73 (21.6)	6 (2.1)	.000
Stretching	71 (21.0)	22 (7.8)	.000
Light strength training	60 (17.8)	14 (5.0)	.000
Moderate strength training	21 (6.2)	9 (3.2)	.081
Leisure			
Golf- carrying bag	1 (.30)	1 (.35)	.898
Golf –using cart	0 (.00)	1 (.35)	.273
Tennis singles	0	0	NA
Tennis doubles	1 (.30)	0 (.00)	.361
Hiking	43 (12.7)	33 (11.7)	.700
Ball sports	15 (4.4)	13 (4.6)	.918
Other			
Mechanic work	23 (6.8)	15 (5.3)	.443
General exercise	49 (14.5)	11 (3.9)	.000
Skating	2 (.59)	0 (.00)	.196
Biking	25 (7.4)	11 (3.9)	.064
Other- not specified	19 (5.6)	5 (1.8)	.013
Accelerometer increase in MVPA (minutes per week)	108 (32.0)	96 (34.0)	.581

^*^ Intent to treat; † between group differences

A logistic regression analysis was conducted for significant activity variables from the bivariate analysis to predict any increase in accelerometer MVPA for the intervention group only are presented in Table 3. There were significant increases in all CHAMPS activities except aerobic machines (O.R. =1.32, *p* = .514) and errand walking (O.R. = 1.50, *p* = .128). Table 3 also shows regression results for logistic regression for decrease in sedentary time. An increase in leisure (O.R. = 2.76, *p* = .046), errand (O.R. = 3.53, *p* = .051), and brisk walking (O.R. =4.784, *p* = .011) were significantly associated with a decrease in sedentary time. Additionally, dance (O.R. = 8.22, *p* = .003), aerobics class (O.R. = 32.7, *p* = .001) and light housework (O.R. =6.75, *p* = .000) were also all associated with a decrease in sedentary time between baseline and follow-up. None of the other variables were significant.

**Table 3:** Odds ratios from logistic regression for an increase in MVPA minutes per week or decrease in sedentary time per day from baseline by increase in activity type of 30 min or greater in the Enlace intervention group (*n* = 338)^a^

	MVPA		Sedentary Time	
	O.R.	*P*-value	O.R.	*P*-value
Walking				
Leisure time	3.22	.000	2.76	.046
Errand	1.50	.128	3.53	.051
Brisk	4.35	.000	4.74	.011
Aerobic exercise				
Dance	2.78	.000	8.22	.003
Aerobic class (Zumba®)	3.66	.000	32.7	.001
Aerobic machines	1.32	.514	.399	.414
Jogging	2.14	.007	1.22	.813
Lifestyle				
Light housework	2.16	.001	6.75	.000
Heavy housework	1.75	.036	1.78	.388
Heavy gardening	1.78	.023	1.25	.682
Light gardening	3.13	.000	1.20	.738
Other				
General exercise	3.21	.000	2.23	.369
Other- not specified	3.25	.015	1.18	.856
Biking	2.49	.029	……	……
Individual Characteristics				
Age	1.36	.016	1.03	.215
BMI	1.03	.009	.988	.638

^a^ Intent to treat

Table 4 presents ANOVA and effect size estimates for each significant activity from the logistic regression analysis. The overall variance explained by this model was 15.6%. Significant effect sizes were observed for jogging (1.2%, *p* = .050), general exercise (1.6%, *p* = .024), and other exercise not specified (2.6%, *p* = .003). Table 5 presents the results for sedentary time. The overall variance explained was 35.3%. Significant effect sizes were detected for leisure time (3.1%), errands (1.7%), brisk walking (2.2%), dance (4.2%), and aerobics class (1.3%). There were no other significant effect sizes observed in this analysis. Furthermore, the contributing variables to explain the variance in MVPA and sedentary time differed completely and there was no overlap for any of the activity types.

**Table 4:** ANOVA and effect size estimates for a change in MVPA minutes per week from baseline by 30 min or greater increase in activity type in the Enlace intervention group (*n* = 338)^a^

	Partial SS	*p*-value	Effect Size (%)
Walking			
Leisure time	20,006.8	.165	.6
Errand	31,281.4	.083	.9
Brisk	133.5	.910	.004
Aerobic exercise			
Dance	681.3	.798	.02
Aerobic class (Zumba®)	7051.6	.409	.2
Aerobic machines	1130.0	.7411	.03
Jogging	39,825.3	.050	1.2
Lifestyle			
Light housework	32,025.6	.079	.9
Heavy housework	9457.8	.339	.6
Heavy gardening	89.1	.926	.002
Light gardening	308.3	.863	.009
Other			
General exercise	53,278.4	.024	1.6
Other- not specified	90,596.3	.003	2.6
Biking	133.5	.9096	.004
Residual	3,316,434.2		
Model % variance explained			15.6

^a^ Intent to treat, adjusted for age and BMI

**Table 5:** ANOVA and effect size estimates for a change in sedentary minutes per day from baseline by 30 min or greater increase in activity type in the Enlace intervention group (*n* = 338)^a^

	Partial SS	*p*-value	Effect Size (%)
Walking			
Leisure time	40,915.4	.036	.031
Errand	491,190.7	.022	.017
Brisk	82,025.6	.003	.022
Aerobic exercise			
Dance	73,597.0	.005	.042
Aerobic class (Zumba®)	63,091.7	.009	.013
Aerobic machines	102.6	.916	.001
Jogging	7046.2	.383	.003
Lifestyle			
Light housework	31,903.3	.064	.013
Heavy housework	5785.5	.429	.001
Heavy gardening	2874.7	.577	.008
Light gardening	17,375.1	.171	.003
Other			
General exercise	10,148.5	.295	.004
Other- not specified	210.9	.880	.000
Residual	2,470,471.2		
Model % variance explained			.353

^a^ Intent to treat, adjusted for age and BMI

## Discussion

The findings from this study demonstrate that certain activity types were associated with increases in MVPA and decreasing sedentary time in Mexican American women from South Texas on the U.S.-Mexico border that participated in this promotora-led PA intervention. Participants who reported at least a 30 min increase in leisure time walking, brisk walking, dance, aerobics classes, jogging, light housework, heavy housework, heavy gardening, light gardening, general exercise, other exercise, and biking had a higher odds of increasing their accelerometer MVPA at follow-up. The effect size analysis however revealed that the type of activities that most contributed to the change in MVPA were jogging, general exercise, and other PA not specified. In addition, this analysis showed that walking, dance and aerobics class increases had significant associations with a reduction in sedentary time and were the only significant activities to contribute to the effect size. These findings suggest that the type of activities planned in PA interventions may influence the success of that program, particularly in populations that are less likely to engage in physical activities.

Few studies have investigated to what extent does type of PA impact the success of an intervention. Those that have, have generally looked at MVPA as their only outcome measure using one measure, usually walking or other aerobic exercise [52]. Success or failure is often attributed to not reaching or maintaining the desired MVPA [53], rather than assessing the concordance between activity type and achievability of sustained behavioral change. Selecting the correct type of activity to obtain the desired outcome may be the key to success. Our study findings provide a unique contribution to the literature by offering this needed comparison of different types of activity options. As a result, we have identified the physical activities most likely to be associated with an increase with MVPA in low income Mexican American women living in a rural setting in South Texas on the U.S.-Mexico border. Although, the participants in our study are low-income Mexican American women, the research is an important first step to better designed interventions that will have greater impact on sustainable behaviors leading to increased MVPA.

Reducing sedentary time has rarely been used as a desired endpoint to PA interventions. This is despite the growing evidence that decreasing sedentary time can have as substantial impact on obesity and disease risk [54–56]. In this study, the physical activities most associated with a decrease in sedentary time were walking, dance and aerobic exercise. Other studies have used increase in walking as the desired outcome in interventions targeted at Hispanics [9]. These studies have demonstrated long-term success in increasing PA, yet have not measured the impact on reducing sedentary time. Future studies are needed to better understand how interventions designed to promote specific physical activities, such as walking, may have a substantial impact on reducing sedentary time.

In the effect size analysis we observed that the greatest PA contributors to changes in MVPA were jogging, general exercise and other- not specified activity. General exercise and other – not specified activity were activities not measured in the CHAMPS instrument and could include activities that are not captured in many instruments. This brings to question whether standard subjective measures of PA are adequately assessing common activities in economically or race/ethnically marginalized group. Some studies have found a general discordance between objective and subjective measures of PA particularly by race/ethnicity [56, 57]. In most occasions, self-reported PA is over estimated relative to objective measurement through an accelerometer [57, 58]. It may be that self-assessed PA measures are not capturing the right type of activities, particularly in marginalized populations with limited resources to be active. For example, there is some evidence to suggest that Hispanic subgroups, like immigrants, may be more likely to walk for utilitarian purposes, but less likely to engage in recreational PA [59] and that duration in the United States has a negative relationship with the amount of walking Hispanic groups engage in [60]. Self-reported instruments, like the CHAMPS may still provide better insight into what types of activities people engage in in general, but may need to be modified to include race/ethnic or rural/urban specific types of preferred activities in order to get a better sense of actual PA and to effectively evaluate the success of intervention programs [61].

### Limitations and strengths

This study has noteworthy limitations. First, our study was conducted in South Texas, on the U.S.-Mexico border. Therefore, the findings can only be inferred to this population. Also, studies would need to be conducted in other groups to corroborate our findings. An additional limitation is our use of the CHAMPS as our measure of subjective PA. The benefit of the CHAMPS is its use of specific types of activities in constructing measurement of PA. However, it is limited in the type of activities and may as a result create some bias in Hispanic or low income populations. Nevertheless there are few, if any instruments available that taking into consideration specific types of activities like the CHAMPS does. Furthermore, there may be other unmeasured factors directly or indirectly responsible for the observed changes in PA. For example, many of the planned PA sessions were in group settings where women also had opportunity to socialize with other women. This may have served as an unmeasured motivation to engage in the physical activities that Enlace offered.

Despite the limitations, the Enlace study was a unique approach to addressing PA deficits in a marginalized race/ethnic minority community. The use of community-based participatory research was key to the design of the intervention and choice of physical activities that women in this community would be most likely to engage in. Additionally, promotoras stressed that increasing activity overall, will have real benefit. This message was reflected in the increase in several types of activities, including housework. Women fit in more activity within their normal routines in their home making increasing MVPA and decreasing sedentary time more achievable. In addition, this study provides an assessment of what type of physical activities can contribute to meaningful changes in PA that impact health and risk of chronic diseases. It also demonstrated what may be realistic expectations for improvements in communities like these with limited resources and built environments not supportive of PA. Mexican American women living in South Texas have limited resources to be physically active, but despite these limitations, were able to incorporate various traditional and non-traditional types of activities in efforts to meet the recommendations. Future studies need to better assess ways in which Mexican American women and other disparate groups are able to achieve recommended levels of PA and how activity is assessed. Understanding how Mexican American women engage in activity will help improve the effectiveness of PA interventions and improve rates of meeting recommendations in groups with low levels of engagement.

## Conclusions

Increasing PA among Mexican Americans and other Hispanic groups is essential to curb current obesity, chronic disease and cancer trajectories. In order to design effective interventions public health practitioners and researchers must take into effect preferences of activities specific to subpopulations like that on the U.S.-Mexico border.

## Data Availability

The data that support the findings of this study are available from the authors on request.

## Abbreviations

BMI: Body Mass Index
CHAMPS: Community health activities model program for seniors physical activity questionnaire
CRC: Community resource centers
MA: Mexican American
MVPA: Moderate-to-vigorous physical activity
PA: Physical activity
SDT: Self-determination theory
